# Molecular Dynamics Simulation Study of Polyamide Membrane Structures and RO/FO Water Permeation Properties

**DOI:** 10.3390/membranes8040127

**Published:** 2018-12-06

**Authors:** Tomohisa Yoshioka, Keisuke Kotaka, Keizo Nakagawa, Takuji Shintani, Hao-Chen Wu, Hideto Matsuyama, Yu Fujimura, Takahiro Kawakatsu

**Affiliations:** 1 Center for Membrane and Film Technology, Graduate School of Science, Technology, and Innovation, Kobe University, 1-1 Rokkodai, Nada, Kobe 657-8501, Japan; 172p106p@stu.kobe-u.ac.jp (K.K.); knakagaw@port.kobe-u.ac.jp (K.N.); shintani@port.kobe-u.ac.jp (T.S.); 2 Center for Membrane and Film Technology, Department of Chemical Science and Engineering, Kobe University, 1-1 Rokkodai, Nada, Kobe 657-8501, Japan; waynewu18@shark.kobe-u.ac.jp (H.-C.W.); matuyama@kobe-u.ac.jp (H.M.); 3 Research and Development Division, Kurita Water Industries Ltd., 1-1 Kawada, Nogi, Shimotsuga, Tochigi 329-0105, Japan; yu.fujimura@kurita.co.jp (Y.F.); takahiro.kawakatsu@kurita.co.jp (T.K.)

**Keywords:** molecular dynamics, polyamide, water permeability, reverse osmosis, forward osmosis

## Abstract

Polyamide (PA) membranes possess properties that allow for selective water permeation and salt rejection, and these are widely used for reverse osmotic (RO) desalination of sea water to produce drinking water. In order to design high-performance RO membranes with high levels of water permeability and salt rejection, an understanding of microscopic PA membrane structures is indispensable, and this includes water transport and ion rejection mechanisms on a molecular scale. In this study, two types of virtual PA membranes with different structures and densities were constructed on a computer, and water molecular transport properties through PA membranes were examined on a molecular level via direct reverse/forward osmosis (RO/FO) filtration molecular dynamics (MD) simulations. A quasi-non-equilibrium MD simulation technique that uses applied (RO mode) or osmotic (FO mode) pressure differences of several MPa was conducted to estimate water permeability through PA membranes. A simple *NVT* (Number, Volume, and Temperature constant ensemble)-RO MD simulation method was presented and verified. The simulations of RO and FO water permeability for a dense PA membrane model without a support layer agreed with the experimental value in the RO mode. This PA membrane completely rejected Na^+^ and Cl^−^ ions during a simulation time of several nano-seconds. The naturally dense PA structure showed excellent ion rejection. The effect that the void size of PA structure exerted on water permeability was also examined.

## 1. Introduction

Membrane separation processes that feature energy saving and ease of handling have been widely utilized to address the problems of clean water shortage and water pollution as global population and economic development both continue to grow. In order to continue answering the problem of increased water demand in the near future, research and development is expected to produce higher-performance separation membranes with excellent water permeability. The practical utilization of such membranes can reduce the footprint of water-treatment plants due to lower requirements for membrane area. Utilization can also contribute to smart solutions to water treatment that will reduce the cost of water production.

Reverse osmosis (RO) membrane separation is representative of methods used to separate solute molecules and water molecules in solute-dissolved aqueous solutions such as in the desalination of seawater. Reasonable water fluxes for seawater of salt concentration greater than 35,000 ppm and brackish water of salt concentration around 1000–5000 ppm are 12–17 LMH/bar (L/m^2^/h) and 12–45 LMH, respectively. Considering the osmotic pressure of seawater and brackish water, the required pressures for desalination are 55–65 bar for seawater and 10–30 bar for brackish water [1]. The main mechanism of water permeation through RO membranes is diffusion and convection flow of dissolved water molecules into the separation layer under pressure [2,3,4,5]. Therefore, preparation of a high performance membrane with both high water permeability and high salt rejection is indispensable for achievement of high water flux. On the other hand, water permeation characteristics depend not only on membrane materials and structures but also on biofouling and scaling. Since, in a long-term RO operation, these factors cause degradation of the membrane desalination performance, development of a fouling-resistant RO membrane is also of importance [1]. Recently, forward osmosis (FO) membrane separation has also attracted attention as a separation method that can save energy and produce an effective concentration of solutes during water production. The relationships between membrane structures and the permeability/rejection performance of RO/FO membranes are still not sufficiently understood compared with those of microfiltration (MF), ultrafiltration (UF), and microporous gas-separation membranes. In order to expand the field of possible applications for membrane separation processes, as fine separation technologies for molecular mixtures of various liquids advances in the future, an understanding of membrane structures and solvent permeation/solute rejection mechanisms on a molecular scale is indispensable. Molecular simulation has shown promise as a tool for studying microscale membrane-related phenomena.

The first molecular dynamics simulation study on RO/FO membrane processes was published in *Chemical Physics Letters* by Murad et al. in 1994 [6]. A semi-permeable membrane was modeled as a single molecular layer composed of 32 Lennard-Jones (LJ) particles connected by virtual springs in a face-centered configuration. The semi-permeability of this membrane was controlled via the spring constant. Two of these membranes covered the sides of a solvent-phase cell, and a two-solution phase cell covered the outer surface of the two membranes. When the density of solvent and temperature was lower, the permeability of solvent particles became higher from solvent to solution, and vice versa. The former could be assumed to correspond to the RO operation and the latter to the FO operation. Despite the simplicity of the simulation method and the small-scale system with an LJ force field, it is astonishing that both the RO and FO permeation modes through semi-permeable membranes were successfully simulated in the 1990s. In the approximately 10 years since, Murad’s group published follow-up papers [7,8], and realistic membrane permeations and membrane materials became diversified in the late 2000s. In 2007, Sridhar et al. calculated nitrogen permeance through polyamide membranes using a unit cell with a highly dense nitrogen region by evacuating the regions on both sides of the membrane [9]. Harder et al. carried out water diffusion simulations using polyamide membranes in 2009 [10], and water flux and salt permeability were evaluated in 2011 [11]. Via the characterization of polyamide microstructures, molecular simulation results were compared with the positron annihilation lifetime spectroscopy (PALS) measurements by Shintani et al. in 2009 [12]. Polyamide structures that included water molecules were also studied for void structures of polyamides and for the density distribution of water molecules by Ding et al. using molecular simulations [13]. In 2015, Ding et al. reported a non-equilibrium molecular dynamics (NEMD) simulation of saltwater (NaCl aq.) under a pressure gradient using close operating conditions in an actual experimental RO system [14]. Following this, the NEMD RO simulation of alcohol under high pressure was examined by Shen et al. in 2016 [15]. Concerning the other membrane materials, RO simulations for boron, nitride and carbon nanotubes [16], silicalite [17], hydrophilic FAU zeolite and hydrophobic MFI zeolite [18] (where FAU and MFI are designations of specific zeolite structures), 2D graphene nanosheet [19,20], and silicon carbide nanotubes embedded in a lipid bilayer [21], have been energetically studied. An NEMD water permeation simulation under a pressure gradient for cellulose triacetate membranes, another major RO membrane material, was reported by Boateng et al. in 2016 [22], but there appears to be no active simulation studies on this material.

In the past, molecular simulations were utilized to evaluate permeability based on the so-called “sorption-diffusion” model of diffusivity and concentration in a membrane under an equilibrium state. In recent years, however, direct molecular simulation methods under non-equilibrium states with long calculation times have more often appeared in the literature, but their quantitative accuracy and adequacy of simulation conditions remain unsatisfactory for an estimation of a more realistic membrane performance.

Papers using more authentic FO molecular simulations have been published since around 2010. Jia et al. carried out FO simulations for saltwater with carbon nanotubes, and demonstrated the optimum nanotube size for high levels of water permeability and salt rejection [23]. In 2015, Song et al. compared the FO performance of porous graphene and carbon nanotubes with different pore sizes, and found that water permeability was higher for a graphene sheet with small pore sizes, but nanotubes with larger pores showed superior water permeability [24]. A channel with a suitable size is a promising candidate for FO membrane material, and, therefore, water and ion transport behavior in water channels composed of spiral protein molecules, such as Aquaporin, have been widely investigated since around 2001 [25,26,27,28]. In those papers, however, water permeability was often calculated based on the diffusive flux across membranes or channels. In 2018, Liu et al. carried out a realistic NEMD FO simulation with an isobaric system of 0.1 MPa across a porous graphitic carbon nitride sheet. In that case, the draw solution (DS) was MgCl_2_ aq. and the feed solution (FS) was NaCl aq. With that setup, water molecules were successfully transported from the FS side to the DS side through the membrane [29]. Wu et al. also staged a successful NEMD FO simulation of water permeation through artificial cyclic peptide channels [30]. As described above, FO simulations have focused on water permeation and salt-rejection properties though porous sheets, nanotubes, and water channels in the development of novel FO membrane materials with extremely high levels of water permeability and salt rejection. FO simulations, however, are not commonly applied to ordinary organic polymer membranes, as far as we could ascertain. When technical issues and calculation costs are considered, however, it is difficult to estimate reliable water permeability given a short amount of time and a small-scale simulation system when the water permeability of a membrane is low. These are among the reasons that FO simulations have seldom been utilized to study important organic polymer membranes with relatively low levels of permeability.

Polyamide membranes have excellent salt rejection properties that make them a practical choice for seawater desalination. Molecular simulation studies looking into the relationships between membrane structure and water permeability/salt rejection have recently increased in number. To obtain a realistic view of the difficulties of permeability due to calculation cost, however, few studies have focused on the water permeability of polyamide membranes per membrane area in comparison to studies of novel channel membranes and direct simulation. Therefore, estimates of water permeability have often been based on diffusivity in bulk polyamide materials [10,11,13,31]. Only recently have direct-permeation simulations of liquids under non-equilibrium conditions begun to emerge [14,15,32]. The objectives of the present study include the modeling of polyamide membrane structures and a realistic evaluation of water permeability. Non-equilibrium FO and RO simulations were carried out for two types of virtual polyamide structures. Correlations between water permeability and polyamide membrane structure were examined on a molecular scale.

## 2. Polyamide Model and Simulation Methods

In the present study, complete modeling and simulation environment software (ver. 2017 R2, BIOVIA Materials Studio^®^, San Diego, CA, USA), was used for all molecular simulations. This software is composed of several modules, and we employed the following modules. Polyamide molecules were constructed in *Visualizer* from monomers, and polyamide membrane models were prepared by handling polyamide molecules in *Amorphous Cell*, which also was utilized for the construction of RO/FO simulation cells as well as in the preparation of saltwater and pure water cells. A Condensed-phase Optimized Molecular Potentials for Atomistic Simulation Studies (*COMPASS*) force field was employed to calculate the interactions and dynamics of molecules. This is the first ab initio force field that has been parameterized and validated using condensed-phase properties with various ab initio and empirical data for isolated molecules [33]. This force field enabled us to perform molecular simulations for accurate prediction of molecule properties such as structure, conformation, vibration, and thermophysics in isolation and in condensed phases. *Forcite Plus* was used for energy calculations, geometry optimizations, and molecular dynamics simulations, and these were the main calculations in the present study. Water adsorption simulations on polyamide models were conducted using a *Sorption* module.

### 2.1. Polyamide Membrane Model

**Figure 1:**
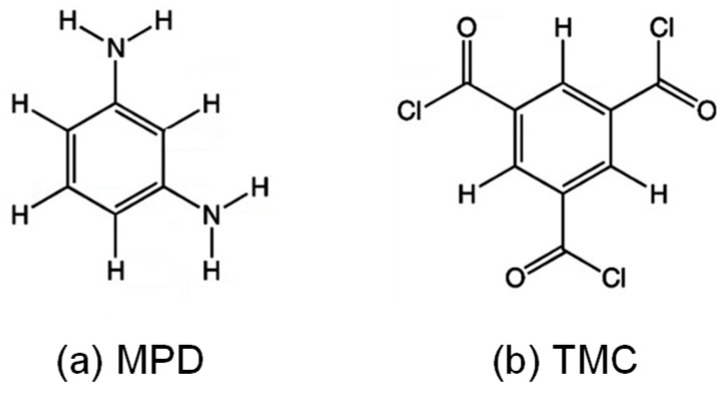
Chemical structure of m-phenylene diamine (MPD) (**a**) and trimesoyl chloride (TMC) (**b**).

A unit of polyamide molecules is composed of two different molecules of m-phenylene diamine (MPD) and trimesoyl chloride (TMC). Figure 1 shows the chemical structures of MPD and TMC [10]. 

**Figure 2:**
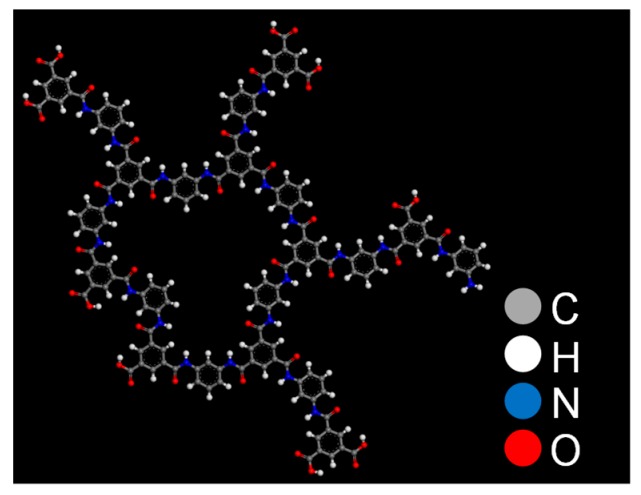
Molecular structure of a polyamide.

The polyamide molecules in this work were composed of 10 MPD and 11 TMC molecules, as shown in Figure 2. Carbon, hydrogen, nitrogen, and oxygen atoms are indicated by the gray, white, blue, and red dots, respectively. This polyamide molecule was composed of 156 carbon, 106 hydrogen, 22 nitrogen, and 39 oxygen atoms. The degree of crosslinking (DC) is defined by Equation (1), where *N*_N_ is the number of nitrogen atoms and *N*_COOH_ is the number of COOH groups [34].

(1) $$\mathrm{DC}=\frac{100N_{N}}{N_{N}+N_{\mathrm{COOH}}}$$

According to Equation (1), with increases in the number of amide bonds, R’-CO-NH-R’’, DC also increases. The DC of the polyamide in Figure 2 was 71.0%, which is very close to the DC of an actual polyamide [34].

**Figure 3:**
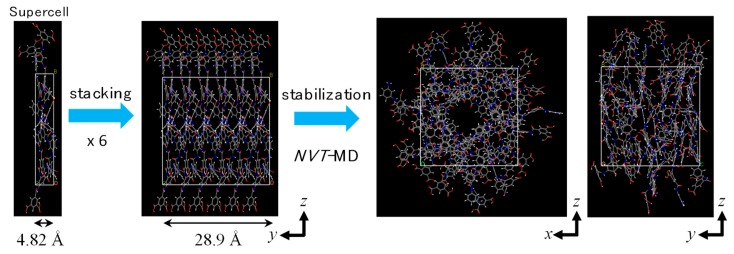
Preparation procedure for a polyamide-1 (PA1) membrane model.

**Figure 4:**
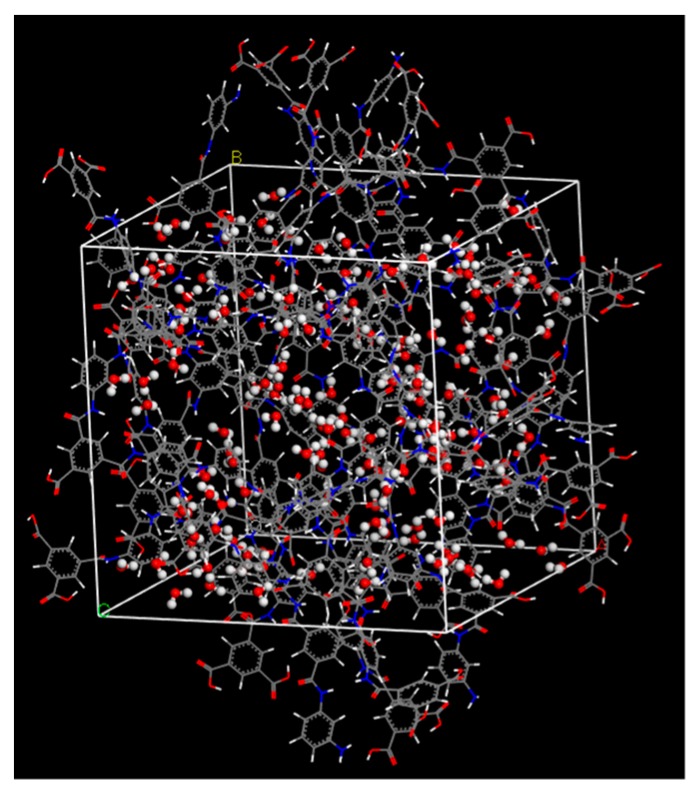
Snapshot of a hydrated polyamide membrane (PA1).

In order to prepare a polyamide membrane structure, 6 polyamide molecules were stacked regularly to a density of 1.2 g/cm^3^ [35]. The procedure used to prepare a PA1 membrane model is shown in Figure 3. First, a supercell was prepared with a thickness one-sixth the length of a final cubic unit cell containing one polyamide molecule. This was repeated six times to form a cubic unit cell that was 1.2 g/cm^3^. Second, each polyamide molecule was rotated around the center of a gravity axis that was perpendicular to a polyamide molecular plane by angles of 0, 270, 315, 135, 90, and 180 degrees, respectively. After geometrical optimization, *NVT*-MD was conducted for 50 ps at 298 K to obtain a stable polyamide structure, polyamide-1 (PA1), where *NVT*-MD was compared with MD simulation under a constant number of molecules (*N*), constant volume (*V*), and constant temperature (*T*) using a canonical ensemble. Using this polyamide structure, water adsorption simulation was conducted under conditions of 100 kPa and 298 K using the Grand canonical Monte Carlo (GCMC) method to obtain a suitable polyamide model saturated by water molecules. Figure 4 shows a snapshot of the PA1 saturated by adsorbed water molecules. There were 143 adsorbed water molecules, and the overall density of the saturated polyamide was 1.376 g/cm^3^, which was within the range of density for actual polyamide materials [35,36].

**Figure 5:**
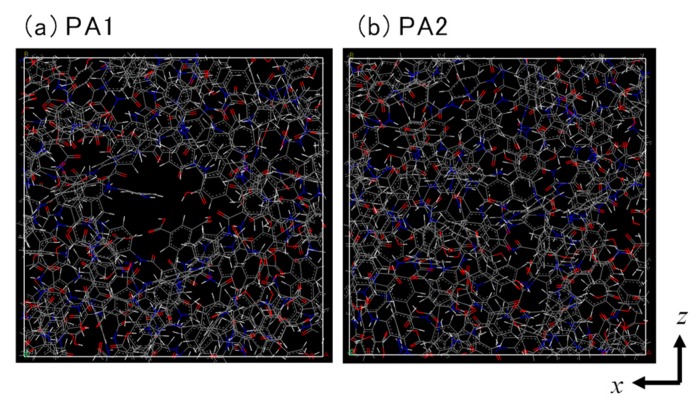
Structure of polyamide-1 (PA1) (**a**) and PA2 (**b**).

**Figure 6:**
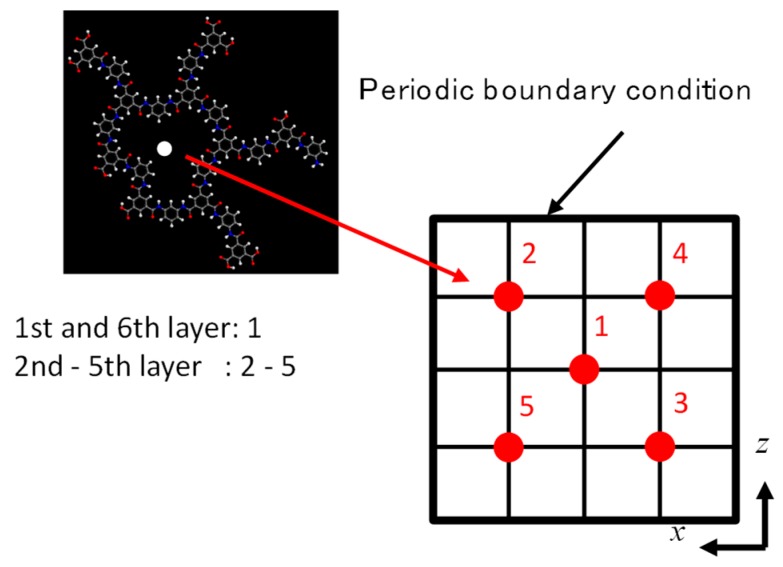
Schematic image of the additional shift of each polyamide layer during preparation of the polyamide-2 (PA2) membrane model.

As shown in Figure 5a, a large void similar to a cylindrical pore was observed in the *z*-direction near the center of the cell for the PA1 model. We also prepared another type of polyamide structure with the same density as that of the PA1 but with more homogeneity. As in the case of the preparation for PA1, after rotation of the stacked polyamide molecules, each polyamide molecule was moved in its *x*-*z* plane to prevent overlapping of their voids through the *y*-direction. This was accomplished when the center white dot in each cyclic polyamide molecule was shifted to the location of the numbered red dot in Figure 6. The centers of the molecules in the first and 6th layers were moved to dot-1, and the 2nd, 3rd, 4th, and 5th layers were moved parallel to dots 2, 3, 4, and 5, respectively. After geometrical optimization, *NVT*-MD was conducted for 50 ps at 298 K to obtain another stable polyamide structure, polyamide-2 (PA2) (Figure 5b). No penetrating pore structures were obvious in the *y*-direction for the PA2 model. Water adsorption simulation was conducted under the same conditions, 100 kPa and 298 K, as PA1 using the GCMC method to obtain PA2 saturation by water molecules. The PA2 model had a water density saturation of 1.373 g/cm^3^ with 113 water molecules. The unit cell size of the PA2 model was somewhat smaller than that of PA1 due to a smaller amount of adsorbed water molecules, but its overall density was within the range of a water-saturated polyamide at 1.37–1.38 g/cm^3^.

### 2.2. FO Simulation Cell

**Figure 7:**
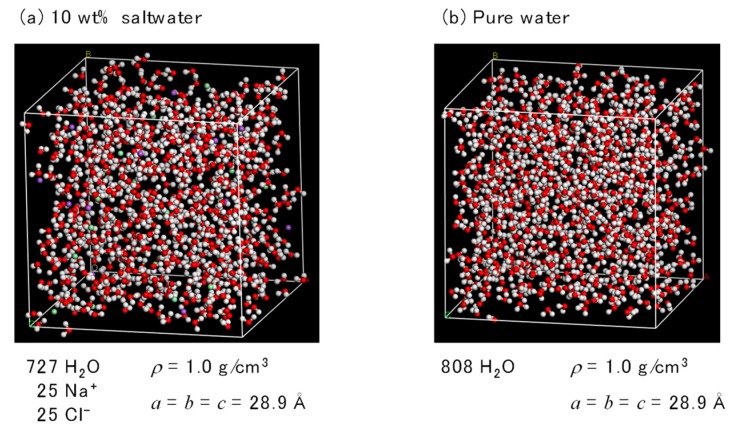
Simulation cells of saltwater (**a**) and pure water (**b**) for polyamide-1 (PA1).

The FO simulation cell was composed of three cells of saltwater, a membrane, and pure water. To simulate PA1, the saltwater and pure-water cells had a cubic size of 28.9 Å^3^, as shown in Figure 7. The concentration of saltwater was set at 10 wt% for a cell that contained 727 water molecules along with 25 Na^+^ and 25 Cl^−^ ions. The pure-water cell contained 808 water molecules. The density of both the saltwater and the pure water was 1.0 g/cm^3^. The osmotic pressure of the saltwater was 8.51 MPa, which was based on the van’t Hoff equation.

The volume for each of the saltwater and pure water cells for PA2 was 28.7 Å^3^. The saltwater cell contained 710 water molecules along with 24 Na^+^ and 24 Cl^−^ ions for a combined concentration of 10 wt%. The pure-water cell for PA2 contained 788 water molecules. The density of both the saltwater and pure water was 1.0 g/cm^3^. Osmotic pressure of the saltwater was 8.37 MPa.

**Figure 8:**
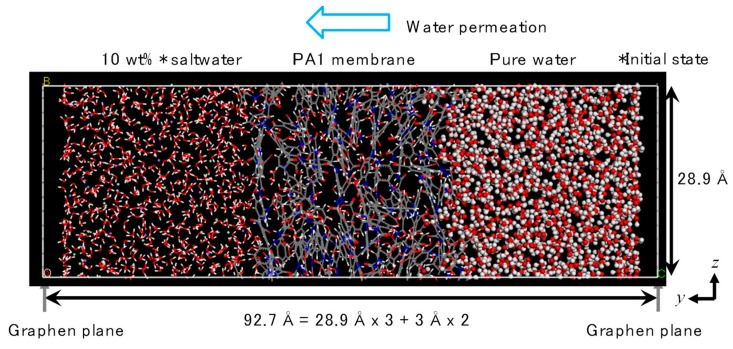
Unit cell for *NVT*-FO simulation (polyamide-1, PA1).

Figure 8 shows an example of the PA1 *NVT*-FO simulation cell. The PA1 was settled at the center part of the simulation cell, which was connected to a saltwater cell on the left-hand side and a pure-water cell on the right-hand side. In order to avoid the mixing of saltwater and pure water at both edges of the simulation cell in the *y*-direction due to the inevitable periodic boundary setting in this software, two graphene planes were settled and fixed at the edges [30]. These graphene planes effectively cut the simulation cell at the edges. A margin of 3 Å between each water and graphene plane was secured at each edge to prevent undesirable compaction and to increase the water density due to repulsive interactions from graphene planes. The PA2 simulation cell was set in the same manner.

**Figure 9:**
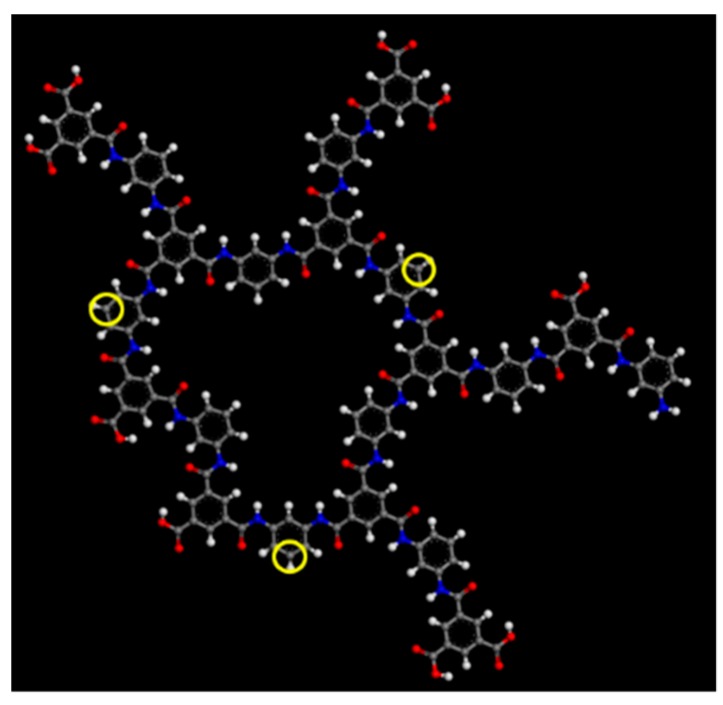
Fixed carbon atoms in a polyamide molecule (marked with yellow circles).

Another small feature of this simulation cell included the fixing of 3 carbon atoms per one polyamide molecule, as shown in Figure 9, to stabilize the membrane. Another geometrical optimization was performed following completion of the construction and setting of the simulation cell, and *NVT*-FO simulation was carried out at 298 K for both the PA1 and PA2 models. The MD calculation required 1 fs. The simulation started from an initial non-equilibrium state with an osmotic pressure difference. Then, by flowing pure water to the saltwater side across the membrane, the osmotic pressure of the saltwater was decreased, the density of the saltwater was increased, and the density of the pure water was decreased. Finally, the osmotic pressure difference and the pressure difference corresponding to the density difference across the membrane were balanced to reach an equilibrated state, where no permeation of water was observed. With this FO simulation method, water permeability can be obtained directly from the water flux at the transition regime by suitably considering the effective pressure difference as a driving force [30].

### 2.3. RO Simulation Cell

**Figure 10:**
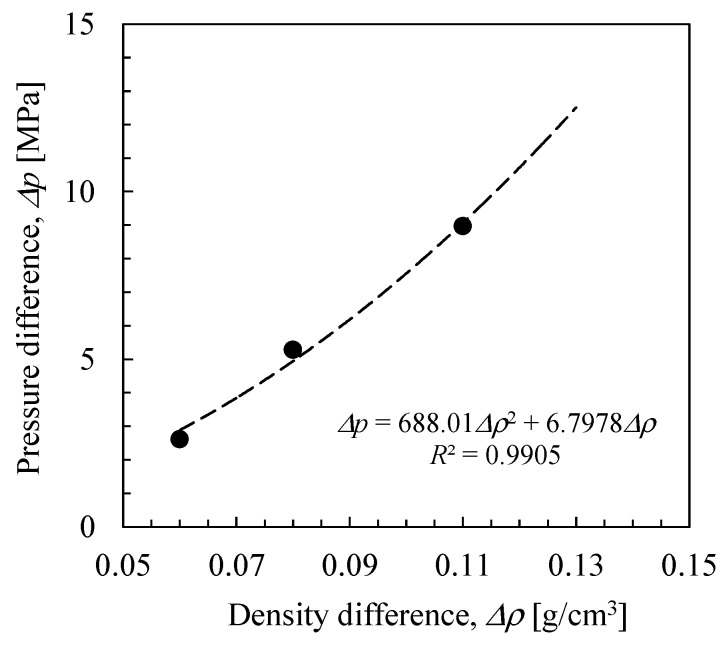
Relationship between pressure difference and water density difference observed in *NVT*-FO simulations.

As mentioned in the previous section, in *NVT*-FO simulation, the transport of water molecules from pure water to saltwater decreased the osmotic pressure of the saltwater and slightly increased and decreased the densities of the saltwater and pure water, respectively. Since the small difference in density corresponded to the pressure difference, the transport of water across the membrane was stopped when the osmotic pressure equaled the pressure difference. Therefore, *NVT*-FO simulations using saltwater cells of different concentrations with several osmotic pressures revealed the relationships between liquid density differences and pressure differences of the system at each equilibrated state where liquid density and osmotic pressure were balanced. Figure 10 shows the relationship obtained from *NVT*-FO simulations when using a cyclic peptide nanotube as a water channel [30].

**Figure 11:**
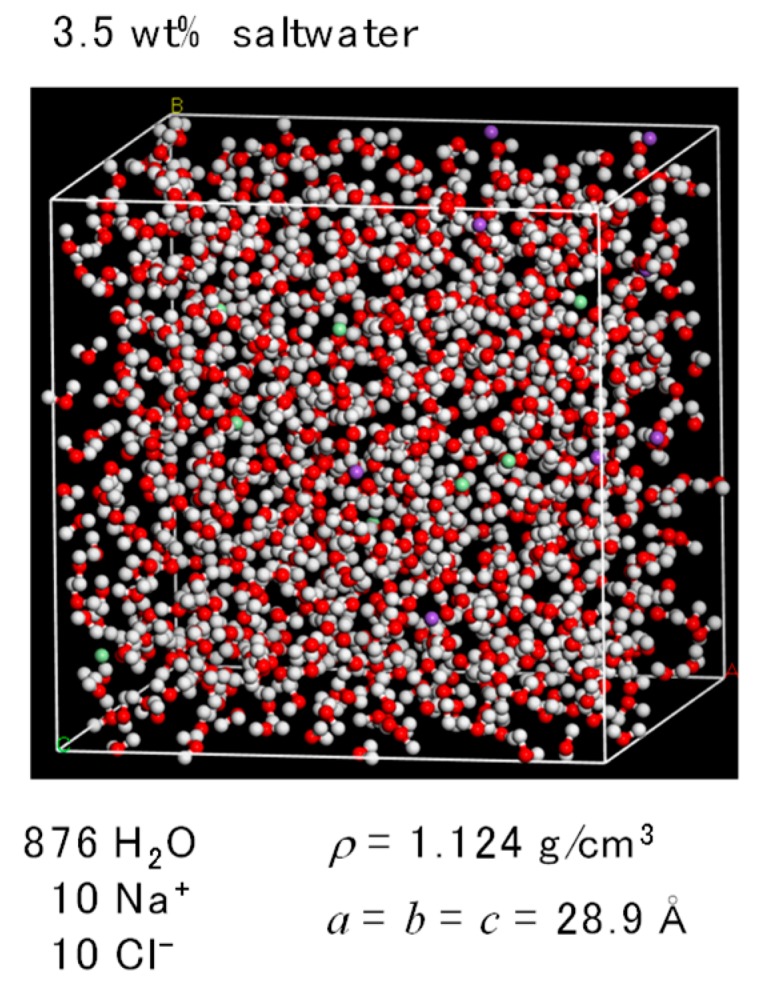
Saltwater cell for *NVT*-RO simulation of polyamide-1 (PA1).

**Figure 12:**
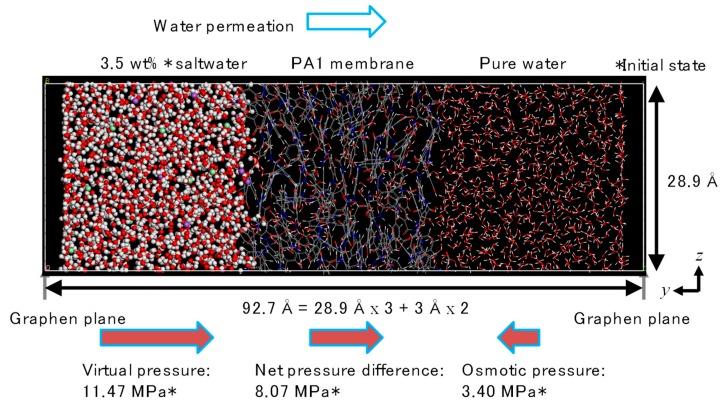
Unit cell for *NVT*-RO simulation (polyamide-1, PA1).

In the present study, the slight differences in the densities of the saltwater and the pure water, as shown in Figure 10, allowed control of the initial amount of virtual pressure to the saltwater side in order to carry out *NVT*-RO simulation under a non-equilibrium state. The concentration of the saltwater was 3.5 wt% and the pressure applied to the saltwater was set at 11.47 MPa for a density difference between the saltwater and the pure water of Δ*ρ* = 0.1242 g/cm^3^, where the saltwater with a *ρ* = 1.1242 g/cm^3^ was composed of 876 molecules of water along with 10 Na^+^ and 10 Cl^−^ ions for the PA1 3.5 wt% saltwater cell shown in Figure 11. The osmotic pressure of this saltwater was 3.40 MPa. The pure-water cell was the same as that used for *NVT*-FO simulation (Figure 7b). Figure 12 shows an *NVT*-RO simulation cell for PA1. The driving force for water permeation was assumed to be the difference between the virtual pressure applied to saltwater, 11.87 MPa, and the osmotic pressure of the saltwater, 3.40 MPa, which totaled 8.07 MPa. Some carbon atoms were fixed and two graphene planes were settled at both edges of the simulation cell, as with *NVT*-FO simulation. For PA2, the cell size was somewhat smaller, and to ensure equal RO simulation conditions, the 3.5 wt% saltwater cell contained 854 water molecules along with 10 Na^+^ and 10 Cl^−^ ions.

## 3. Results and Discussion

### 3.1. Polyamide Structure

**Figure 13:**
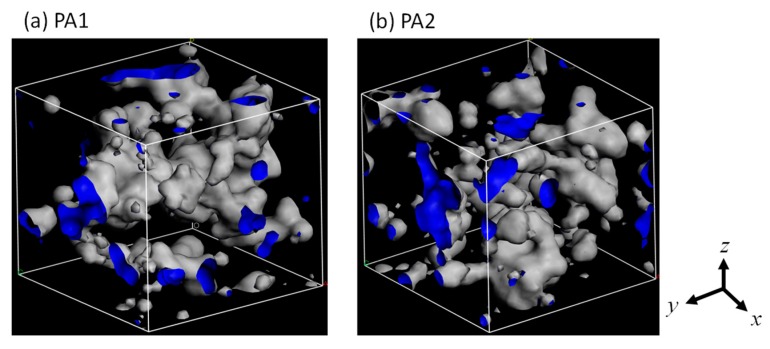
Snapshots of the void volumes of polyamide-1 (PA1) (**a**) and PA2 (**b**).

Figure 13 shows snapshots of the void structures of PA1 and PA2, as measured using a probe sphere with a diameter of 0.3 nm, which approximated that of the water molecule. PA1 had a relatively large void through the central part of the cell in the *y*-direction. The PA2 had smaller voids that were uniformly distributed throughout the cell. The porosities using a 0.3 nm sphere for PA1 and PA2 were 19.5% and 16.8%, respectively, which indicated that PA1 would possibly allow for a more effective void and transport of water molecules.

**Figure 14:**
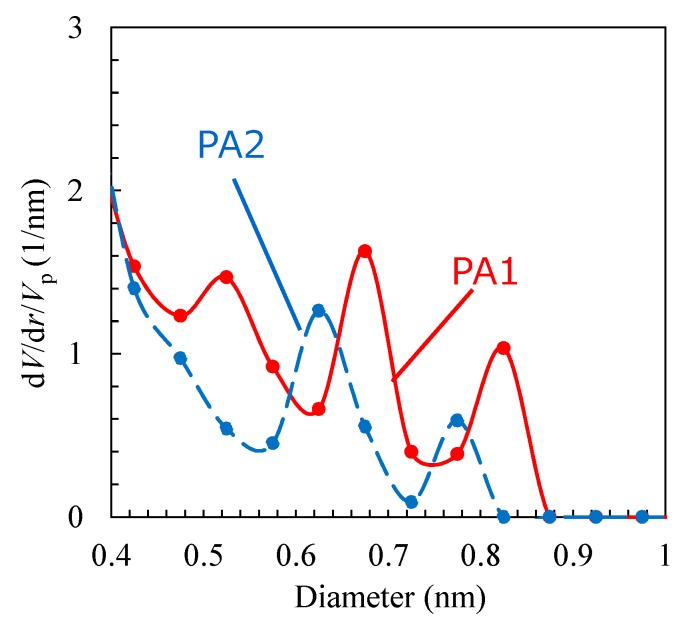
Void distribution of polyamide-1 (PA1) and PA2.

The void-volume distributions of these two models are shown in Figure 14, as calculated by the “Grid point in L-sphere” method [37]. This technique enables a quantitative correction of the evaluation of very small voids by checking the size of the largest sphere in which each grid point of a void is included. Both the polyamide models had no pinholes greater than 0.9 nm. PA1, however, had somewhat larger voids that were assumed to have come from the space around the center region of the cell, as shown in Figure 5a. On the other hand, the PA2 model had a void of approximately 0.6 nm, which is very close to the dominant void size of the polyamide material, as measured by PALS [38,39]. Therefore, PA2 exhibited a more reasonable typical polyamide structure. The structural characteristics of the PA1 and PA2 membrane modes are summarized in Table 1.

### 3.2. NVT-FO Simulation

**Figure 15:**
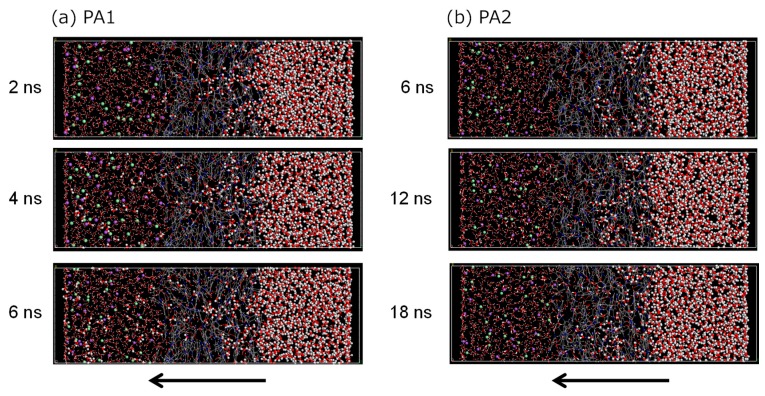
Snapshots of *NVT*-FO simulation for polyamide-1 (PA1) (**a**) and PA2 (**b**).

Figure 15a,b shows snapshots of the *NVT*-FO simulations for the PA1 and PA2 membrane models, respectively. In each image, water molecules located on the pure-water side (right-hand side of the membrane) at the initial state are indicated by the ball shapes, and the water molecules on the saltwater side and in the membrane are represented by the stick and rod shapes, respectively. The purple and green balls on the saltwater side represent the Na^+^ and Cl^−^ ions, respectively. For the PA1 model, the center region of the membrane was effective for the rapid transport of water molecules, which had already reached the saltwater side at 4 ns. For PA2, however, no pure-water molecules had permeated the saltwater region even after 6 ns, but an intrusion of many water molecules into the membrane was clearly observed. As pure-water molecules intruded into the PA2 membrane, water molecules in the membrane seemed to be transported into the saltwater side as if the water molecules had pushed them out from the pure-water side. The water permeability of PA2 was obviously lower than that of PA1. Ion leakage to the pure water was not observed for the PA1 nor the PA2 models during the simulation time.

**Figure 16:**
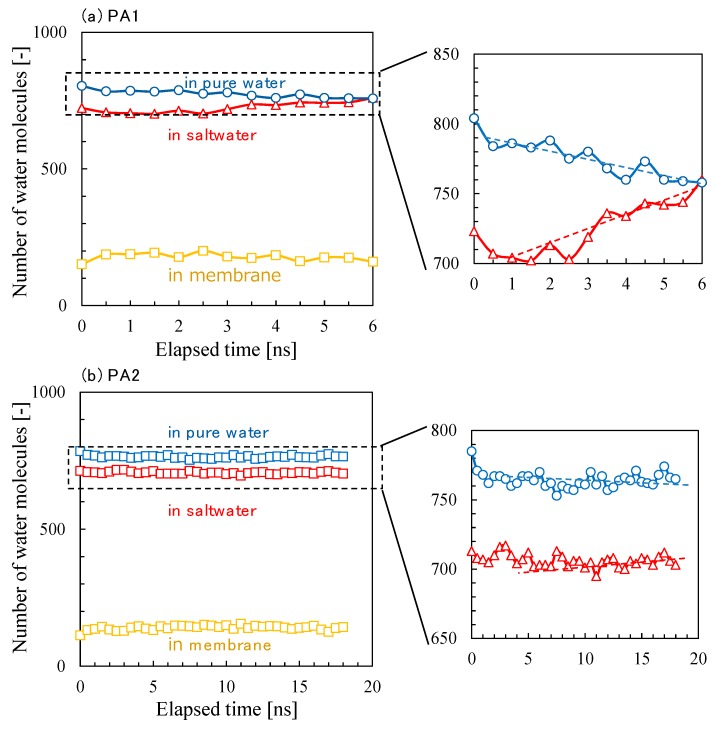
Time courses for the numbers of water molecules in pure water, saltwater, and polyamide (PA) membranes during *NVT*-FO simulations for PA1 (**a**) and PA2 (**b**).

Time courses for the number of water molecules in each cell during the *NVT*-FO simulation are shown in Figure 16. The number of water molecules in the membrane was nearly constant during the simulation, while that of the pure-water cell had decreased and that of the saltwater cell had increased with time for both the PA1 and PA2 models. In particular, the number of water molecules on both sides of the PA1 membrane had substantially changed, which indicated that the water permeability of PA1 was higher than that of PA2. In both simulations of PA1 and PA2, the transport of water molecules from the pure water to the saltwater was apparent, which suggests that the FO permeation simulation was successful.

**Figure 17:**
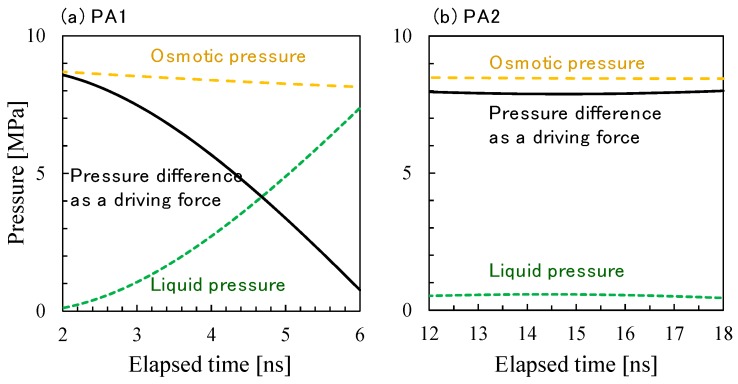
Time courses of osmotic pressure differences, liquid pressure differences, and pressures as the driving force of water permeation during *NVT*-FO simulations for polyamide-1 (PA1) (**a**) and PA2 (**b**).

The slope of the change in the number of water molecules with time can be used to estimate the water permeation flux. The driving force for permeation is the difference between the osmotic pressure of saltwater and the liquid pressure difference corresponding to density difference. Figure 17 shows the osmotic pressure of saltwater calculated using the van’t Hoff equation, virtual pressure (= liquid pressure difference) estimated from the relationship shown in Figure 10, and the pressure difference as a net driving force for water permeation as a function of elapsed time. The FO water permeability, *P*_FO_ [LMH/bar (L/m^2^/h/bar)] was estimated using the data in Figure 16 and Figure 17, *P*_FO_ (PA1) = 350–400 LMH/bar and *P*_FO_ (PA2) = 15–25 LMH/bar. It should be noted that these values are for the membrane models with a thickness of approximately 3 nm, whereas the thickness of an actual polyamide membrane is several times than that of these virtual membranes. Due to fluctuation in the change in the number of water molecules and to a very small amount of slope, the error range would not be considered small, but the calculated water permeability of the PA2 membrane model seemed comparatively reasonable [40]. The water permeability of PA1, however, was too large for a true comparison to a polyamide RO membrane. The through-hole void structure, approximately 0.8–0.9 nm as shown in Figure 14, of the PA1 membrane model would cause a very high level of permeability. Slight differences in the major void size of RO membranes can have a large influence on water permeability.

### 3.3. NVT-RO Simulation

**Figure 18:**
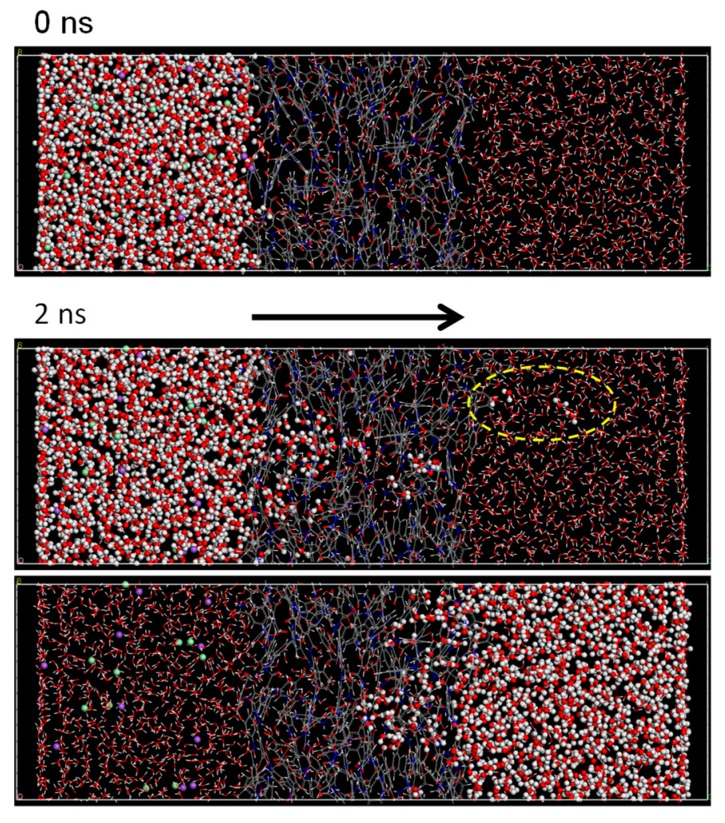
Snapshots of *NVT*-RO simulation for polyamide-1 (PA1).

Figure 18 shows a snapshot of the *NVT*-RO simulation for the PA1 membrane model. In order to simplify the viewing of the transport of water molecules, in the upper two images in Figure 17, water molecules located on the saltwater side (left-hand side of the membrane) at the initial state (at 0 ns) are indicated by ball shapes and water molecules in the pure-water side and in the membrane are shown as stick shapes. After 2 ns had passed, water molecules moved from the saltwater to the pure-water side, which was contrary to the results of *NVT*-FO simulation. The image at the bottom in Figure 17 is the same as the middle image, but the appearance of the water molecules is reversed (saltwater: stick shape; pure water: ball shape). The bottom image shows no water molecules being transported from the pure water to the saltwater side at 2 ns.

**Figure 19:**
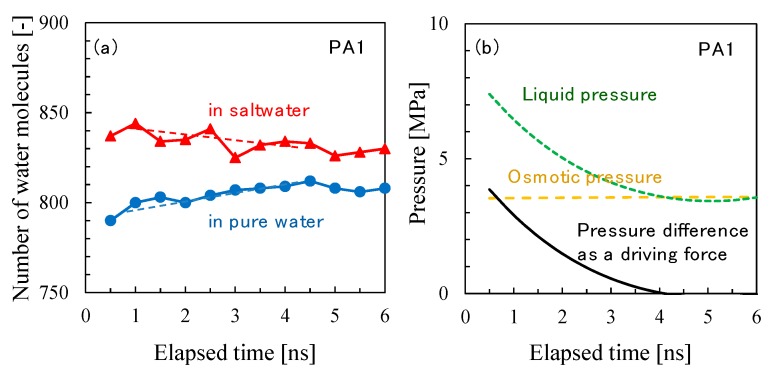
Time course of the number of water molecules in pure water and saltwater (**a**) and the osmotic pressure difference, liquid pressure difference, and pressure as a driving force of water permeation (**b**) during *NVT*-RO simulation for polyamide-1 (PA1).

Time courses for the number of water molecules in each cell during the *NVT*-RO simulation are shown in Figure 19a. Unlike the *NVT*-FO simulation in Figure 16, the number of water molecules in the pure-water region increased and that in the saltwater region decreased with time. The transport of water molecules from the saltwater side to the pure-water side shows that RO permeation simulation was successful.

**Figure 20:**
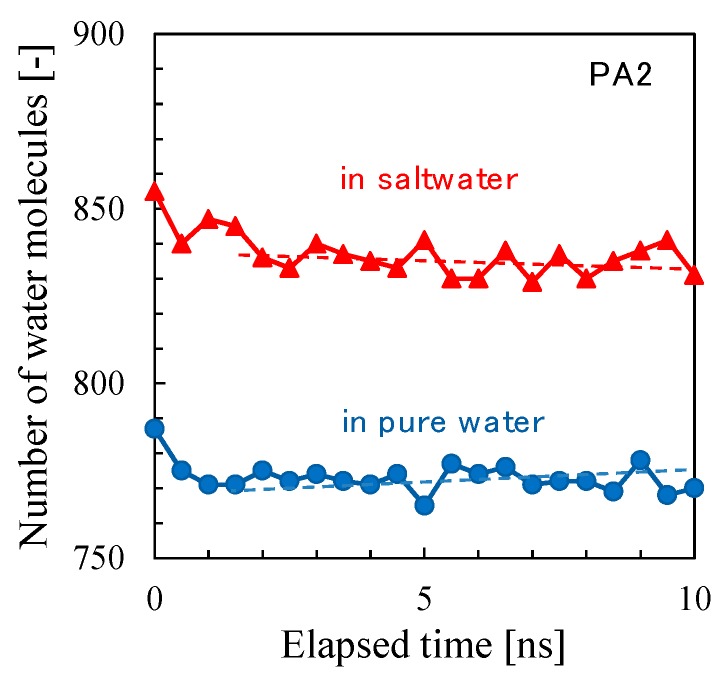
Time course of the number of water molecules in pure water and saltwater during *NVT*-RO simulation for polyamide-2 (PA2).

Figure 19b shows the osmotic pressure difference, liquid pressure difference, and pressure as a driving force of water permeation as a function of elapsed time during the *NVT*-RO simulation for PA1. The change in the net pressure difference across the membrane as a driving force for water permeation largely depended on either the trend in liquid pressure or the change in density difference. The RO water permeability estimated from Figure 19 was *P*_RO_ (PA1) = 550–600 LMH/bar. This value is about 1.5 times higher than that for *P*_FO_ (PA1), however, considering the accuracy of these simulations, it is apparent that the water permeability estimated for the different permeation simulation modes of *NVT*-FO and *NVT*-RO were sufficient for comparison. The change in the number of water molecules during the *NVT*-RO simulation of the PA2 membrane model is shown in Figure 20. In the RO simulation mode, the observed water permeation flux for PA2 was smaller than that for PA1, which was similar to the results of the FO simulation mode. The RO water permeability estimated for PA2 was *P*_RO_ (PA2) = 4–5 LMH/bar. The accuracy of this value should be carefully examined. However, the PA2 model gave a more realistic result for water permeability compared with that from the PA1 model, even in RO simulation mode, which underscores the reliability of this *NVT*-RO simulation method and also that of the PA2 membrane model.

All calculated water permeabilities of the polyamide membrane models for RO and FO simulation modes are summarized in Table 1. In comparison with the actual RO membrane water permeabilities reported in a previous study [41], the PA1 model showed a water permeability that is apparently too high, and it was also not a realistic polyamide model. On the other hand, the water permeability of the PA2 model showed good agreement with that of low pressure-RO membranes. Therefore, the PA2 model seemed to have a membrane performance corresponding to an RO membrane that can be utilized for the desalination of brackish water. We did not observe salt permeation during both the RO and FO simulation period. This PA model might have high salt rejection, but we could not calculate the salt permeability quantitatively. The calculation of salt permeability and rejection from molecular simulations should be important future work.

## 4. Conclusions

The PA2 membrane model had voids of approximately 0.6 nm and showed a lower, but more realistic, level of water permeability than that of the PA1 membrane model, which had slightly larger voids, both in the FO and RO simulation mode. The models of polyamide presented in this work were not validated by comparison with experimental structural data of actual polyamide membranes. Even PA2 still needed to be improved to describe more realistic features of polyamide membranes. Due to the limitation of simulation cost, a membrane model with a large number of molecular weights and with enough membrane thickness cannot be utilized. The limitation of simulation time also made it difficult to obtain the ion rejection properties by using the PA models and the simulation method. It should be noted that these are limits of our model. In this work, instead of directly pressurizing the saltwater, the virtual pressure difference was a driving force that was obtained by establishing the density difference between two liquid regions across a membrane, and *NVT*-RO simulation where the water molecules permeated a polyamide membrane from saltwater to pure water was successful. This simplified RO MD simulation technique differed from that of conventional non-equilibrium MD under a chemical potential gradient that is strictly controlled and kept constant. Therefore, the accuracy of the obtained permeability should be carefully considered. Using our method, however, more easily allows estimates of water permeability in the RO mode compared with conventional NEMD simulation. By employing sufficiently large simulation cells and enough calculation time, the presented RO simulation method would enable more reliable estimates of permeability when using molecular simulation software for general purposes with no specific difficulties.

## Figures and Tables

**Table 1:** Features and performances of polyamide-1 (PA1) and PA2 membrane models.

	Major Void Size (nm)	Porosity (%)	*P*_FO_ (LMH/bar)	*P*_RO_ (LMH/bar)	Salt Leakage
PA1	0.65–0.85	19.5	350–400	550–600	Not detected
PA2	0.58–0.72	16.8	15–25	4–5	Not detected
